# Supplementing Genistein for Breeder Hens Alters the Growth Performance and Intestinal Health of Offspring

**DOI:** 10.3390/life13071468

**Published:** 2023-06-28

**Authors:** Mingkun Gao, Jiao Wang, Zengpeng Lv

**Affiliations:** State Key Laboratory of Animal Nutrition, Department of Animal Nutrition and Feed Science, College of Animal Science and Technology, China Agricultural University, Beijing 100193, China

**Keywords:** maternal effect, genistein, intestinal health, offspring, microbiome

## Abstract

**Simple Summary:**

This essay highlights that genistein supplementation in breeder hens improved the intestinal health of their offspring. The results showed increased birth weight and serum protein levels in chicks, with improved intestinal villus morphology. This effect lasted until 21 days. Maternal genistein also protected against LPS-induced damage and dysfunction by upregulating tight junction proteins. The 16S rRNA gene sequencing revealed an enhanced abundance of *Escherichia coli* and reduced *Gammaproteobacteria* in offspring. In summary, maternal genistein can improve the gut microbiota and intestinal barrier in chicken offspring.

**Abstract:**

Recent research revealed that dietary genistein supplementation for breeder hens can improve the immune function of offspring chicks. However, it remains unknown whether this maternal effect could improve the intestinal health of offspring. This study was conducted to explore the mechanism involved in the maternal effect of genistein on the intestinal mucosa and microbial homeostasis of chicken offspring. A total of 120 Qiling breeder hens were fed a basal diet, a 20 mg/kg genistein-supplemented diet, or a 40 mg/kg genistein-supplemented diet for 4 weeks before collecting their eggs. After hatching, 180 male offspring (60 chickens from each group) were randomly selected and divided into three groups: (1) the offspring of hens fed a basal diet (CON); (2) the offspring of hens fed a low-dose genistein-supplemented diet (LGE); (3) the offspring of hens fed a high-dose genistein-supplemented diet (HGE). At 17 d, 72 male offspring (48 chickens from CON and 24 chickens from LGE) were divided into three groups: (1) the offspring of hens fed a basal diet (CON); (2) the CON group challenged with LPS (LPS); (3) the LGE group challenged with LPS (LPS + LGE). The results showed that maternal genistein supplementation increased the birth weight and serum level of total protein (TP), followed by improved intestinal villus morphology. Continuously, the maternal effect on the body weight of chicks lasted until 21 d. Additionally, it was observed that maternal genistein supplementation exhibited protective effects against LPS-induced morphological damage and intestinal mucosal barrier dysfunction by upregulating the expression of tight junction proteins, specifically *ZO-1*, *Claudin1*, *E-cadherin*, and *Occludin*, at 21 d. Using 16S rRNA gene sequencing, we demonstrated that maternal supplementation of genistein has the potential to facilitate the maturation of newly hatched chicken offspring by enhancing the abundance of *Escherichia coli*. Additionally, maternal genistein supplementation can effectively reduce the abundance of *Gammaproteobacteria*, thus mitigating the risk of bacterial diversity impairment of LPS. In light of these findings, maternal genistein supplementation holds promise as a potential strategy for ameliorating intestinal mucosal damage and modulating the microbiome in chicken offspring.

## 1. Introduction

Intestinal inflammation is common among broiler chickens during their intermediate growth stages. This inflammatory response can be attributed to a range of factors, including the presence of lipopolysaccharide (LPS) [1,2]. In addition, the immunity of chicks with delayed growth in their initial developmental phases deserves attention [3]. The immature immune function of the intestinal system could potentially elevate their vulnerability to pathogenic microorganisms. The ensuing intestinal inflammation may cause considerable harm to the intestinal barrier. This can result in compromised functionality of the intestinal epithelium and a decrease in nutrient absorption capability [4]. This could result in decreased growth performance and significant financial losses in poultry production [5]. It is therefore crucial to explore strategies for chickens’ intestinal development and function. The maternal nutritional strategy plays a critical role in regulating animal offspring phenotypic traits. Proper maternal nutrition is vital for early embryonic development, and studies have shown that the nutritional manipulation enhanced offspring’s internal immunity [6]. Nevertheless, the potential impact of maternal supplementation with flavone extract intervention on chicken offspring remains to be elucidated.

Genistein (GEN), a type of isoflavone, is abundant in soybean products. It can improve the broiler’s growth performance and immune function [7]. It is noteworthy that genistein undergoes primary hydrolysis in the ileum of the maternal body before being transported to the liver via blood circulation. GEN may directly enter the ovary in hens due to the physiological link between the liver and ovary [8,9]. Our previous study shows that genistein supplementation in breeder hens could promote offspring growth and regulate immune function by influencing the hepatic RNA expression profile [8]. In addition, dietary GEN supplementation for breeders and their offspring can improve chickens’ growth performance by regulating the Toll-like receptor signaling pathway [10]. GEN can also alter lipid metabolism in the offspring through epigenetic modification and improve the antioxidative capability [11]. However, the research investigating the effects and mechanisms of perinatal genistein consumption on transgenerational intestinal health through vertical transmission of maternal nutrition and maternal microbiota remains unknown.

It has recently been suggested that the gut microbiota can be vertically transmitted from the mother to her infant [12], according to the microbiome composition of maternal hens’ feces, embryos, and chicks’ ceca. A significant correlation has been shown between the microbiomes and a set of core genera possibly vertically transmitted from the hen’s intestine to the chick has been defined [13]. The nutrients contained within eggs play a crucial role in the early growth and development of chickens. It has been posited that alterations to the maternal diet can influence the nutrient composition of eggs, thereby impacting the nutritional status and intestinal microbiota of chicken offspring [14]. In recent studies, dietary GEN supplementation altered the gut microbiota and increased short-chain fatty acid concentrations [15]. However, whether the GEN intake experienced by breeder hens could regulate the gut microbiota in offspring is unclear. Modifications to the gut microbiota of maternal hens may have an impact on the microbial communities present in chicken offspring. A transgenerational study on GEN supplementation in mothers and their offspring will be of considerable interest.

This study aims to investigate the maternal effects of GEN, a dye derived from Dyers Woad, on offspring development. Specifically, we hypothesize that the presence of GEN in the maternal diet can alter the early colonization of offspring microbiota, leading to subsequent changes in gut health during later stages. By examining the potential impact of GEN on offspring microbiota structure, this research seeks to provide valuable insights into the role of maternal factors in shaping the microbiota composition and its implications for long-term gut health.

## 2. Materials and Methods

### 2.1. Animals and Treatments

A total of 180 Qiling breeder hens were randomly divided into three groups: control, 20 mg/kg genistein-supplemented group, and 40 mg/kg genistein-supplemented group at 60 weeks age.

In a controlled experimental environment, hens were fed a basal or genistein-supplemented diet for four weeks. Genistein (Kai Meng. Co. Xi An, China) Chemical Plant with a purity of 99.8% was added to the basal diet (NY/T33–2004) (Table 1) at the expense of an equal amount (20 mg/kg and 40 mg/kg) of limestone to balance the total nutrient composition of the diets. During the final two weeks of the experiment, all breeder hens underwent artificial insemination. Subsequently, during the experiment’s final week, 60 eggs were collected from each group. The incubation process was performed using the same incubator for all hatched eggs. Following hatching, 240 one-day-old male chickens (comprising 80 individuals from each group) were relocated to breeding cages situated within a temperature- and light-controlled room with continuous illumination. The ambient temperature was maintained at 33–35 °C for the initial week before being gradually reduced by increments of 1 °C every two days until reaching a final temperature of 26 °C. All chicken offspring had ad libitum access to the same diet and water. The composition of the diet for the chicken offspring is presented in Table 1. The basal diet was in accordance with the Chinese Feeding Standard of Chicken (NY/T815-2004). Three groups of chickens were then randomly divided into 8 replicates, and each replicate contained 10 chickens: (1) the offspring of hens fed a basal diet (CON); (2) the offspring of hens fed a low-dose genistein-supplemented diet (LGE); (3) the offspring of hens fed a high-dose genistein-supplemented diet (HGE). The growth performance exhibited by the HGE group was deemed suboptimal. Consequently, a randomized sampling method was employed to select 72 chickens from both the CON and LGE groups, with the intention of administering LPS injection at 17 days post-hatch: (1) the offspring of hens fed a basal diet (CON); (2) the CON group challenged with LPS (LPS); and (3) the LGE group challenged with LPS (LPS + LGE). The body weight and total feed consumption were recorded to calculate the growth performance of the CON, LGE, and HGE groups at 1 and 21 d. Generally, LPS from Escherichia coli (L2880, Sigma-Aldrich Inc., St. Louis, MO, USA) was dissolved in a 0.9% sterile saline solution. According to the method of Lv [16], the LPS injection was performed at 6:00 a.m. at 17, 19, and 21 d. The dose of LPS was 1 mg kg^−1^, whereas the CON group received an injection of sterile saline.

### 2.2. Sample Collection

To ensure a humane method of euthanizing the chicken offspring, the birds in our study were intravenously injected with sodium pentobarbital at a dosage of 50 mg/kg body weight. Serum was subsequently separated and stored at −20 °C. The ileum was carefully extracted, and at 21 days post-injection of LPS, one chicken was randomly selected from each replicate (comprising eight chickens per group). The selected chickens were euthanized, and the ileum was carefully extracted. Each ileum was cut into 2 cm sections, fixed in 4% paraformaldehyde, and preserved for analysis. Additionally, approximately one gram of ileal chyme at 21 days and meconium were collected for DNA extraction.

### 2.3. Serum Biochemical Assays

An array of commercial regent kits (Nanjing Jianchceng Bioengineering Institute, Nanjing, China) was used to determine the TG, cholesterol, glucose, total protein, and albumin levels in serum.

### 2.4. Histological Measurements

Following a 24 h fixation period, ileum specimens underwent dehydration in ethanol and clearance in xylene before being embedded in paraffin. Using a Leica RM2235 microtome (Leica Biosystems Inc., Buffalo Grove, IL, USA), sections were cut at a thickness of 5 μm. To observe and determine intestinal morphology, sections were deparaffinized with xylene and rehydrated through a graded series of ethanol before being stained with hematoxylin and eosin. Each chicken was represented by an experimental unit comprising from 6 well-formed villi.

### 2.5. Total RNA Extraction and mRNA Quantification

The ileum was collected, snap frozen in liquid nitrogen, and stored at −80 °C. According to the method of Li [17], the mRNA extraction, quality control, reverse transcription, and real-time quantitative analysis were performed. Specifically, 0.1 g of ileum was added to a centrifuge tube containing 1 mL of Trizol (Invitrogen Life Technologies, Carlsbad, CA, USA) and vortexed thoroughly. The total RNA was extracted using a kit (Takara, Dalian, China) and subsequently assessed for quality. Reverse transcription was carried out using an M-MLV cDNA kit (Invitrogen Life Technologies), and the resulting product was diluted by a factor of four before being subjected to real-time polymerase chain reaction (RT-PCR) using an Applied Biosystems 7500 Fast Real-Time PCR System (Foster City, CA, USA). The genes’ primer sequences are detailed in Table 2.

### 2.6. Sequencing of the 16S Ribosomal RNA (rRNA) Gene

In this study, a subset of fifteen samples comprising meconium and ileal chyme was randomly selected, with five samples taken from each of the respective groups. The 16S ribosomal RNA (rRNA) gene was sequenced for the purpose of gene analysis. The total genomic DNA was extracted following the method of Li [17]. Sequencing libraries were generated using the Ion Plus Fragment Library Kit (Thermo Scientific, Wilmington, DE, USA), and the final library was sequenced on an Ion S5 XL platform (Thermo Scientific, Wilmington, DE, USA).

### 2.7. Data Analysis of the 16S rRNA Gene

In line with the quality-controlled process implemented by Cutadapt (version 1.9.1), high-quality clean reads were obtained subsequent to data filtration on the raw reads. Uparse software (version v7.0.1001) was employed to analyze the sequences, with sequences exhibiting a similarity of ≥97% being assigned to the same operational taxonomic units (OTUs). Subsequently, we assessed alpha and beta diversity based on the output normalized OTU abundance information data. The Chao1 index was used to estimate the abundance of the bacterial community, while the Shannon indices were applied to evaluate its diversity. To examine the beta diversity of meconium and ileum microbiota, principal coordinate analysis (PCoA) was carried out. Further, we performed linear discriminant analysis effect size (LefSe) to determine the differentially abundant bacteria between the LPS and LSTE groups. The LefSe software (http://huttenhower.sph.harvard.edu/galaxy/root?tool_id=lefse_upload, accessed on 12 February 2020) performs linear discriminant analysis (LDA) on samples according to different grouping conditions based on taxonomy to identify communities or species that have a significant differential impact on sample partitioning. Additionally, functional pathways were predicted in the Kyoto Encyclopedia of Genes and Genomes (KEGG) databases using Tax4Fun functional prediction based on the 16S rRNA gene sequences.

### 2.8. Statistical Analysis

A total of eight replicates of chicken offspring were evaluated for growth performance, and gene expression was measured in six randomly selected samples. Data of body weight, serum biochemical and morphological analysis, and gene expression were statistically analyzed by one-way ANOVA. T-tests were performed to analyze the meconium’s data of 16S rRNA. Data are presented as the mean and standard error of the mean (SEM). The difference was considered to be statistically significant at *p* < 0.05.

## 3. Results

### 3.1. Growth Performance

**Figure 1:**
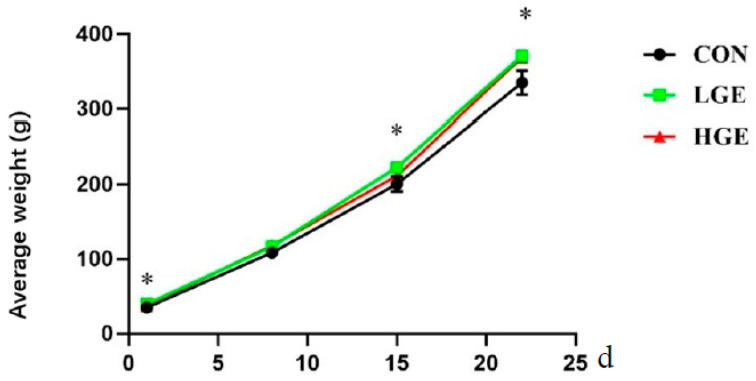
Effects of maternal genistein supplementation on the average weight of chicken offspring. Data are presented as mean values ± SEM (n = 8). Values denoted with an asterisk indicate a statistically significant difference. * for 0.05 > *p*-value.

As shown in Figure 1, maternal supplementation with LGE significantly increased the hatching weight at 1, 7, 14, and 21 d (*p* < 0.05). However, over a period of 21 days, chicken offspring with HEN did not show any difference in growth performance (*p* > 0.05).

### 3.2. Serum Biochemical Indices

The effects of GEN in the diet of breeding hens for 4 weeks on the serum indices of 1-day-old male offspring are shown in Table 3. The addition of 20 mg/kg and 40 mg/kg GEN to the diet significantly reduced the serum levels of UA and CREA in 1-day-old male offspring (*p* < 0.05); both low and high GEN increased this parameter compared to the control group (*p* < 0.05) and increased the serum levels of TP. There were no significant effects on other serum indices.

### 3.3. Intestinal Morphology

**Figure 2:**
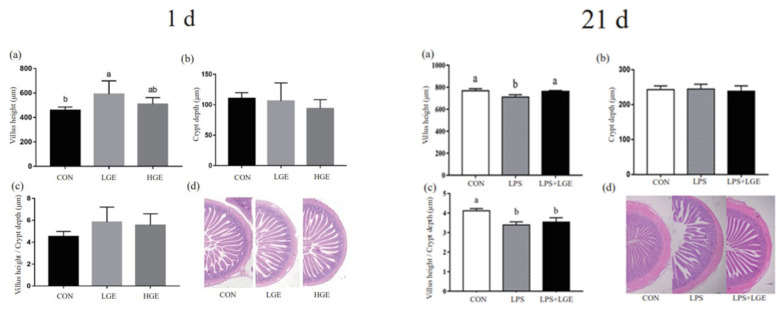
Effect of maternal genistein supplementation on the ileum morphology of chicken offspring: (**a**–**c**) villus height, crypt depth, villus height-to-crypt depth ratio of ileum in the chicken offspring at 1 and 21 d; (**d**) the representative images of HE staining on the ileum.

A hematoxylin–eosin staining was performed to observe how GEN supplementation and LPS challenge affected the intestinal morphology of offspring chickens (Figure 2). The feeding of GEN to breeding hens significantly increased the ileum villus height of 1-day-old broiler offspring, with the most significant effect at 20 mg/kg (*p* < 0.05); however, there was no significant effect on crypt depth or the villus height/crypt depth ratio. Compared to the control group, LPS caused the ileum villus to be broken and disarranged, and morphological damage was observed. The injection of LPS significantly reduced the villus height (*p* < 0.05), whereas maternal GEN supplementation and LPS injection had no effect in the intestinal morphology (*p* > 0.05)

### 3.4. Intestinal Gene Expression

**Figure 3:**
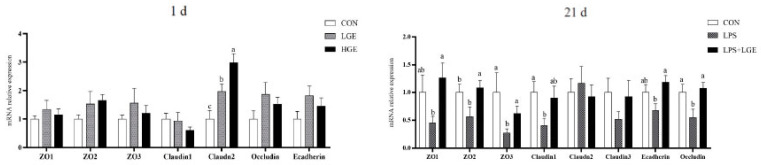
The influence of maternal genistein supplementation on gene expression in the ileum of chicken offspring. Data are presented as mean values ± SEM.

Figure 3 demonstrates that the different levels of maternal GEN supplementation resulted in an increase in the mRNA expression levels of the offspring 1-day-old broiler ileum tight-junction-related genes *ZO2* (*p* = 0.081), *Claudin2* (*p* < 0.05), and *Occludin* (*p* = 0.097), as well as the mRNA expression level of the adhesion-linked gene *E-cadherin* (*p* = 0.091). At the age of 21 d, LPS challenge decreased mRNA expression levels of ileal tight junction genes *ZO1*, *ZO2*, *ZO3*, and *Claudin1* and tight-junction-protein-related proteins *Occludin* and adhesion junction protein *E-cadherin* (*p* < 0.05). However, maternal supplementation significantly increased the mRNA expression of *ZO1*, *ZO2*, *ZO3*, *Claudin1*, *Occludin*, and *E-cadherin* (*p* < 0.05).

### 3.5. Description of the 16S rRNA Gene Sequencing Data

**Figure 4:**
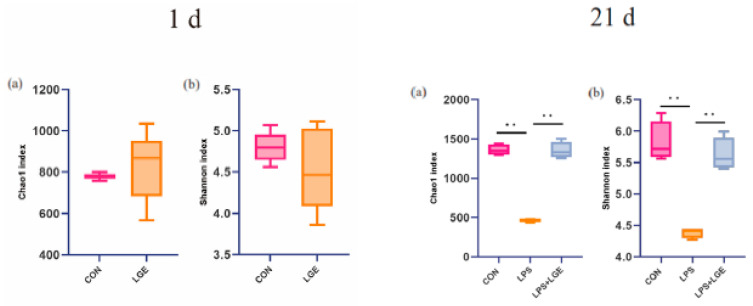
Alpha diversity of meconium and ileum chyme microbiota: (**a**) the Chao1 index; (**b**) the Shannon index. Data represent mean values ± standard error of the mean (n = 5); ** for 0.05 > *p*-value.

**Figure 5:**
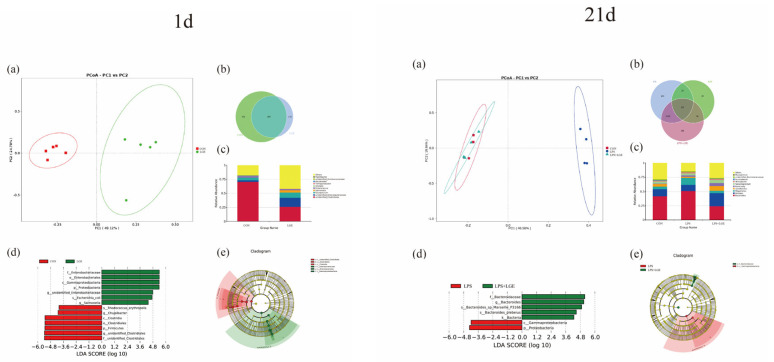
Analysis of the gut microbiome in the chicken offspring: (**a**) PCoA analysis at 1 and 21 d; (**b**) Venn diagram of OTUs at 1 and 21 d; (**c**) the outcome of the ten most prevalent bacterial genera; (**d**) variations in gut microbiota structures as determined by LEfSe analysis at 1 and 21 days; (**e**) biomarkers of the different groups at 1 and 21 d.

The 16S rRNA gene sequencing was conducted on both the meconium and ileal chyme samples obtained from chicken offspring at 1 and 21 d of age, respectively. An assessment of alpha diversity is presented in Figure 4. No significant differences were observed in the 1 d meconium among the groups. LPS challenge significantly decreased the number of Chao1 and Shannon indices (*p* < 0.05). The principal coordinate analysis (PCoA) of the operational taxonomic units (OTUs) demonstrated a distinct separation between the meconium samples of the LGE group and those of the CON group. The PCoA of OTUs demonstrated that the ileum microbiota of the LPS group at 21 days was distinctly separated from both the CON and LPS + LGE groups (Figure 5). A Venn diagram analysis conducted at 1 day of age revealed that there were 695 overlapping OTUs between the CON and LGE groups (Figure 5). At 21 d of age, the Venn diagram revealed that 557 OTUs were overlapped among the CON, LPS, and LPS + LGE groups (Figure 5).

### 3.6. Structure of the Meconium and Ileum Microbiota

Figure 5 presents the top 10 most abundant bacterial genera in both the meconium and ileum chyme samples. At 1 day of age, maternal supplementation with genistein resulted in a marked reduction in the relative abundance of unidentified Clostridiales in meconium when compared to the CON group. The LGE group exhibited a significant increase in the abundance of unidentified *Enterobacteriaceae* and *Epulopiscium* compared with the CON group. At 21 d of age, compared with both CON and LPS + LGE groups, the LPS reduced the relative abundance of *Alistipes*. The LPS + LGE group displayed a markedly elevated prevalence of *Lactobacillus* and *Barnesiella.*

We further performed the LefSe analysis to distinctive bacteria between the CON and LGE groups, LPS, and LPS + LGE groups (Figure 5d). In the group of newly hatched chicken offspring that received LGE treatment, there was a higher relative abundance of *Enterobacteriaceae*, *Gammaproteobacteria*, unidentified *Enterobacteriaceae*, and *Escherichia coli* observed, whereas it had reduced abundance of *Clostridia, Firmicutes*, and *Clostridiales*. In the chicken offspring at 21 d, when compared to both the CON and LPS groups, the LPS + LGE group had a higher relative abundance of *Bacteroidaceae*, *Bacteroides*, *Bacteroides plebeius*, and *Bacteria*. In the LPS group, the proportions of both *Gammaproteobacteria* and *Proteobacteria* were observed to be higher.

### 3.7. Predicted Functional Alterations of Microbial Communities

**Figure 6:**
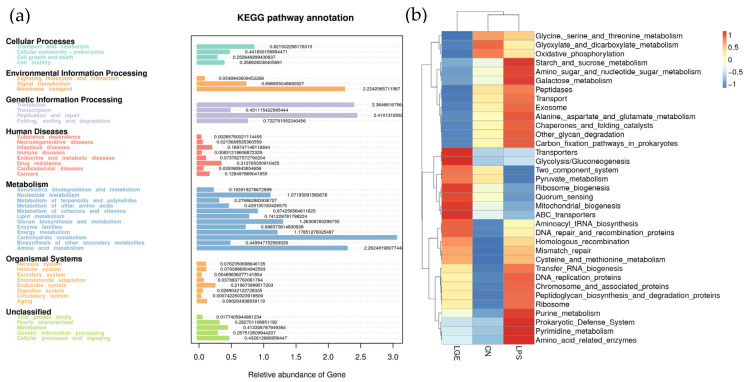
Tax4Fun prediction of gut microbiota function in the chicken offspring at 21 d: (**a**) KEGG pathway annotation; (**b**) prediction of gut microbiota function clustering heat map.

The composition of gut microbiota is closely associated with intestinal health. As depicted in Figure 6, the Tax4Fun analysis was employed to evaluate the predicted functional changes within the microbial communities. The KEGG functional clustering result shows that the enriched pathways had strong connections with transport and catabolism in cellular processes, membrane transport in environmental information processing, translation and replication and repair in genetic information processing, and carbohydrate metabolism and amino acid metabolism in metabolism. Maternal supplementation of GEN improved ileum microbial energy metabolism, the endocrine system, and immune system disorders in broilers caused by LPS challenge; however, it inhibited the intestinal microbial lipopolysaccharide protein and lipopolysaccharide synthesis processes and the NOD-like signaling pathway. In addition, it promoted fatty acid catabolism, as well as provided good alleviation of neurodegenerative diseases, immune diseases, environmental adaptation, and circulatory system and digestive system functions.

## 4. Discussion

LPS challenge has been shown to induce intestinal mucosal damage and alter the gut microbiome [18]. Our study suggested that maternal GEN supplementation could promote the average body weight of chicken offspring and alleviate LPS-induced intestinal damage. The specific manifestations are improved intestinal morphology, mucosal immune function, tight junction, and gut microbiota.

The observed increase in hatching weights in chicken offspring suggests that the administration of genistein supplements to the mother may have a beneficial influence on the embryonic development of her offspring. The birth weight of the chicks plays a crucial role in determining the growth rate during the broiler stage and is closely associated with the overall growth performance during the slaughter period [19]. However, due to the characteristics of the local chicken breed, the quality of the offspring chicks of Qiling Grass Chicken breeding hens in the late egg-laying period gradually declines, and the growth rate can be reduced. Research confirms that nutritional measures in breeding birds can regulate the growth and development of offspring through material deposition and epigenetic modifications [20,21]. GEN has a “programmed” effect on the growth and development of mammalian offspring [22]. GEN can significantly improve embryonic development parameters (embryo length and embryo weight) in ROSS broiler offspring, promote skeletal muscle development (breast muscle rate and muscle fiber diameter) in the broiler stage, and then improve growth performance [8]. In poultry production, adding GEN to diets can increase broiler feed intake and body weight gain, reducing the FCR [23]. Supplementing hens with GEN can alter lipid metabolism in the offspring through epigenetic modification and improve growth performance. Therefore, it is feasible to regulate offspring development by adding GEN to breeder diets [11]. Our results show that maternal supplementation of 20 mg/kg GEN could increase the 1~21 d growth of chicken offspring. Similarly, earlier research suggests that maternal supplementation of 400 mg/kg GEN and offspring fed 40 mg/kg GEN could significantly increase the chickens’ body weight gain and decrease the FCR of female broilers during 1~21 d [11]. Previous studies have suggested that the maternal effects of GEN primarily enhance offspring productivity by promoting skeletal muscle development. However, our study posits that the maternal effects of GEN primarily influence offspring growth and development by promoting early intestinal development.

The intestine serves as a crucial site for nutrient absorption. It is important to note that during early chick development, the rate of intestinal development surpasses the rate of body weight growth [24]. Nutritional intake in the early growth phase is particularly significant, as it determines the growth bottleneck for chicks. For optimal growth and development in chicks, it is critical to maintain proper intestinal villus morphology, as this is related to the capacity for nutrient digestion and absorption. A higher villus-to-crypt ratio (VCR) is generally regarded as a sign of superior epithelial turnover. Newly hatched chicks undergo early digestive system development to support growth and development during the broiler fattening stage [25]. Our intestinal morphological results demonstrate that maternal LGE supplementation significantly increases the villus height of 1-day-old chicks, as well as the ratio of villus height to crypt depth in the intestine. This indicates that supplementation of maternal GEN can enhance nutrient absorption in offspring by promoting early intestinal development, leading to increased average body weight in the offspring.

Given the rapid circulation of intestinal epithelial cells, significant energy and protein consumption is required to maintain the swift production of new cells [26]. Serum urea and protein levels are direct indicators for evaluating the protein metabolism of the organism. In this experiment, GEN’s effect significantly reduced the serum urea level and increased the protein level in the offspring at 1 d. This suggests that the maternal GEN effect can promote the protein synthesis process and inhibit protein catabolism in offspring.

The intestinal physical barrier refers to the structural components and mechanisms that protect the intestinal lining and regulate the passage of substances into and out of the gut. It plays a crucial role in maintaining intestinal health. One of its key functions is the regulation of nutrient absorption. The barrier selectively permits the absorption of nutrients, ensuring optimal uptake for the body while preventing the uptake of harmful substances. This regulation helps maintain a healthy balance in nutrient absorption within the intestine [27]. GEN has been found to reduce intestinal stem cell depletion and improve intestinal epithelial regeneration disorders, thereby enhancing intestinal epithelial barrier function [28]. *Claudin-2*, a tight junction protein belonging to the claudin family, is a primary component of the intestinal barrier [29]. Increased transcription levels of Claudin-2 in the LGE and HGE groups provided evidence of improved intestinal integrity after maternal GEN supplementation. This also serves as key evidence for improving gut health in the offspring chicks.

The challenge of lipopolysaccharide (LPS) has been demonstrated to elicit several physiological responses, including hyperemia, bleeding spots, alterations in gut microbiota composition, and damage to the intestinal mucosa [30]. Consequently, LPS infection has been found to reduce the absorption capacity of the intestines. However, the current study demonstrates that the addition of GEN to the diet of broilers can protect against LPS-induced morphological injury in the ileum, suggesting that it confers benefits to the intestinal barrier [16,31]. The comparison of villus height between the LPS and LPS + LGE groups may be attributed to a superiorly developed intestinal structure resulting from maternal GEN supplementation. Moreover, tight junction proteins, including *Occludin*, *ZO-1, ZO-2, ZO-3, Claudin*, and *E-cadherin*, play a significant role in regulating intestinal barrier function, contributing to the maintenance of intestinal integrity [32]. The reduced mRNA expression of *ZO-1, ZO-2, ZO-3, Claudin1, Occludin*, and *E-cadherin* in response to the LPS challenge indicates that it can impair intestinal integrity. Thus, maternal GEN supplementation can improve the intestinal barrier function of chickens. This may be attributed to the critical role played by early GEN in the early intestinal development of chicks, enhancing the integrity of the intestinal barrier and reinforcing gut health and thereby countering the damage caused by LPS.

The transfer of microorganisms from the maternal body to the offspring is a crucial mechanism for the formation and development of a robust initial microbiome. This process has substantial implications for infant growth and the maturation of their immune system [33]. The gut microbiome’s stability and resilience are critical ecological characteristics that affect host health. Research suggests that microbial colonizers present in early embryos may have been inherited from maternal hens. During development, environmental factors and host genetic variation can influence gut microbial abundance and diversity [13]. Essential elements, such as the diversity of microorganisms, the ability to adapt metabolically, the presence of redundant functions, and the interactions between microorganisms and between microorganisms and their host, play a vital role in sustaining resilience [34]. The present investigation reveals that maternal genistein supplementation has the potential to exert a significant impact on the microbiota of neonatal chickens, ultimately leading to the modulation of the gut microbiome of the chick. A prior study demonstrated a noteworthy increase in the gut microbial population during the development of chicken embryos from E3 to E12 [35]. The augmentation of the gut microbial population observed during embryonic development potentially correlates with the establishment of diverse organs and systems, such as the endocrine and immune systems, within the early-stage chicken embryo [35].

Our study demonstrated a significant increase in the abundance of *Escherichia coli* on the first day of hatching, which also happened to be the most abundant member of the newborn’s gut microbiota [36]. This observation is likely to reflect a decrease in oxygen concentration and a more defined ecological niche. In the later embryonic stages, the egg’s energy supply mode is primarily based on the oxidation of fatty acids and the gluconeogenic pathway in the liver and muscles, which consumes a significant amount of oxygen. Further evidence supports the notion that facultative aerobes initially colonize the chicken gut, which is later replaced mainly by facultative anaerobes at the late phase [37]. This substitution may be one of the significant reasons why the abundance of *Enterobacteriaceae, Gammaproteobacteria,* and *Escherichia coli* significantly increased in the LGE group. Therefore, anaerobic bacteria (i.e., *Escherichia coli*) and parthenogenic anaerobes have been shown to react to chick development. This reaction might promote newly hatched chickens’ growth and potentially maintain microbial homeostasis [38].

The modification of microbial populations in the ileum serves as a critical marker for the gut’s nonspecific response. The gut microbiota can produce LPS to modulate immune system function [39]. However, LPS infection can disrupt the homeostasis of the ileum microbiota, as evidenced by the significant changes in the alpha and beta diversity of bacterial communities observed following LPS challenge. In contrast, the alpha and beta diversity of the ileum microbiota in the LPS + LGE group exhibited similarities to those of the CON group, indicating that maternal genistein supplementation potentially improves intestinal integrity and mitigates gut microbiota perturbations induced by LPS challenge. At the genus level, the LPS + LGE group exhibited a higher relative abundance of *Lactobacillus* and *Alistipes* compared to the other two groups. These are beneficial bacteria that can play vital roles in promoting digestive health and overall well-being in broiler chickens [40]. Studies have demonstrated that *Lactobacillus* can stimulate broilers’ immune systems, helping them against infections and diseases [41]. Moreover, the linear discriminant analysis effect size (LEfSe) revealed that the LPS + LGE group had a lower *Gammaproteobacteria* population than the LPS group. This decrease in *Gammaproteobacteria* abundance may have contributed to the improvement of intestinal inflammation. In agreement with these findings, it was observed that maternal GEN supplementation modulated the gut microbiome, which may enhance intestinal immune function. It is important to acknowledge that the presence of genistein in egg yolk has the potential to affect the microbiota of chicken offspring, and the supplementation of genistein could also lead to changes in the gut and oviduct microbiota of breeder hens. We hypothesize that these alterations may subsequently influence the microbiome composition in the offspring. However, additional investigations are necessary to validate this novel discovery.

In order to highlight the alterations in the gut microbiota, we conducted a Tax4Fun analysis to forecast the functional outcomes induced by maternal GEN supplementation in chickens subjected to LPS challenge. The microbiota within chicks exhibits an increased abundance of available functions involved in metabolic pathways related to infectious diseases, energy metabolism, and the digestive system. A lower degree of divergence in metabolic pathways between chicks and maternal hens suggests that their gut microbiome performs similar functions, despite differences in their gut microbial composition. These results were obtained from an evaluation of microbial community and functional capacity across various developmental stages and host breeds.

## 5. Conclusions

In summary, our findings suggest that maternal supplementation with GEN may mitigate intestinal mucosal injury and regulate the gut microbiome in chicken offspring challenged with LPS. This novel approach presents a promising strategy for augmenting poultry’s overall health and well-being.

## Figures and Tables

**Table 1:** Ingredient compositions and nutrient levels of the basic corn–soybean diet.

Items ^1^	Breeder Hens	1 to 21 d
Ingredient (%)		
Corn (CP 7.8%)	54.99	53.28
Soybean meal (CP 43%)	34.15	38.57
Soybean oil	0.50	3.70
Limestone	7.23	1.05
Dicalcium phosphate	2.09	1.98
DL-Methionine	0.17	0.17
NaCl	0.35	0.35
^1^ Trace element premix	0.30	0.50
^2^ Vitamin premix	0.10	0.10
Choline chloride (50%)	0.12	0.30
Total	100.00	100.00
Nutrient levels		
Metabolizable energy (MJ /Kg)	11.84	12.35
Crude protein (%)	16.10	21.57
Available phosphorus (%)	0.47	1.15
Calcium (%)	3.48	1.05
Methionine (%)	0.34	0.49
Lysine (%)	0.81	1.05

^1^ Trace element premix (provided per kilogram of feed) of the following substances: copper, 8 mg; zinc, 75 mg; iron, 80 mg; manganese, 100 mg; selenium, 0.15 mg; iodine, 0.35 mg. Calculated value based on the analysis of experimental diets. ^2^ Vitamin premix (provided per kilogram of feed) of the following substances: vitamin A, 12,500 IU; vitamin D3, 2500 IU; vitamin K3, 2.65 mg; vitamin B1, 2 mg; vitamin B2, 6 mg; vitamin B12, 0.025 mg; vitamin E, 30 IU; biotin, 0.0325 mg; folic acid, 1.25 mg; pantothenic acid, 12 mg; niacin, 50 mg.

**Table 2:** Primers used for quantitative real-time PCR analysis.

Gene	Primer Sequence (5′→3′)	Amplicon Size (bp)	GenBank Accession Number
E-cadherin	F: AGCCCCAGTGCTTCTCTCTA	197	NM_131820.1
	R: CCTCCCGATCAGCAACTCTC		
Claudin-1	F: ACCCACAGCCTAAGTGCTTC	200	NM_001013611.2
	R: AGGTCTCATAAGGCCCCACT		
Claudin-2	F: AGGGGCTATGGATGGAGTGT	189	NM_001277622.1
	R: AGCCCTGATTGAAGACGGTG		
Claudin-3	F: AGGGGTTCTCAGCTCTCACT	332	NM_204202.1
	R: GTTTCTCCGCCAGACTCTCC		
Claudin-5	F: TTGCAGGTCGCCAGAGATAC	269	NM_204201.1
	R: AGGCAAGTGCATGTTACCGA		
ZO-1	F: ACTGTGACCCCAAAACCTGG	294	XM_015278981.2
	R: CTCCCTGCTTGTGGCATGTA		
ZO-2	F: GGCTCCCAAAATGAGATGCG	123	NM_204918.1
	R: TTGGGCGTGACGTATAGCTG		
ZO-3	F: CACAAAGGGTTACGCAAGGC	198	XM_015299760.2
	R: AGAGCTCCAGGAGGGTCTTC		
Occludin	F: ACCAGAATGGTACCCTGAGC	141	XM_025144247.1
	R: ATTACACAGCTTCAGCCTTACA		
GAPDH	F: TCAGCACCATCTGACAATGC	148	NC_006088.5
	R: AGTAGGCAGCATCCCATCTG		

F: represents forward; R: represents reverse.

**Table 3:** Effect of maternal genistein on serum indicators of 1-day-old broilers.

Indexes	CON	LGE	HGE	*p*-Value
UA (μmol/L)	995 ± 147 ^a^	621 ± 56.8 ^b^	673 ± 94.1 ^b^	0.035
CREA (μmol/L)	21.6 ± 1.30 ^a^	17.2 ± 0.86 ^b^	14.7 ± 1.62 ^b^	0.006
GLU (mmol/L)	9.60 ± 0.17	8.93 ± 0.42	8.27 ± 0.46	0.134
TCHO (mmol/L)	9.47 ± 0.05	9.31 ± 0.19	9.28 ± 0.15	0.623
TG (mmol/L)	1.59 ± 0.21	1.74 ± 0.21	1.56 ± 0.12	0.770
TP (g/L)	26.6 ± 0.92 ^b^	29.7 ± 0.71 ^a^	31.9 ± 2.41 ^a^	0.024

The data in the table are expressed as mean ± standard error (n = 8). The mean without the same mark (^a, b^) indicates a statistically significant difference (*p* < 0.05). UA, uric acid; CREA, creatinine; GLU, glucose; TCHO, total cholesterol; TG, triglyceride; TP, total protein.

## Data Availability

Data is available upon request from the authors.
